# The quantification of sodium intake from discretionary salt intake in New Zealand using the lithium-tagged salt method

**DOI:** 10.3389/fnut.2022.1065710

**Published:** 2023-01-19

**Authors:** Nan Xin Wang, Rachael M. McLean, Claire Cameron, Sheila A. Skeaff

**Affiliations:** ^1^Department of Human Nutrition, University of Otago, Dunedin, New Zealand; ^2^Department of Preventive and Social Medicine, University of Otago, Dunedin, New Zealand; ^3^Biostatistics Centre, University of Otago, Dunedin, New Zealand

**Keywords:** sodium, salt, discretionary salt, New Zealand, 24-h urine, lithium-tagged salt

## Abstract

**Introduction:**

Discretionary salt (added in cooking at home or at the table) is a source of sodium and iodine in New Zealand. The amount of discretionary salt consumed in a population has implications on policies regarding sodium and iodine. Sodium intake from discretionary salt intake has not been quantified in New Zealand. The aim of this study was to estimate the proportion of total sodium that comes from discretionary salt in adults using the lithium-tagged salt method.

**Methods:**

A total of 116 healthy adults, who were not pregnant or breastfeeding, regularly consume home-cooked meals and use salt during cooking or at the table, aged 18–40 years from Dunedin, New Zealand were recruited into the study. The study took place over a 9-day period. On Day 1, participants were asked to collect a baseline 24-h urine to establish their normal lithium output. From Day 2 to Day 8, normal discretionary salt was replaced with lithium-tagged salt. Between Day 6 and Day 8, participants collected another two 24-h urine samples. A 24-h dietary recall was conducted to coincide with each of the final two 24-h urine collections. Urinary sodium was analysed by Ion-Selective Electrode and urinary lithium and urinary iodine were analysed using Inductively Coupled Plasma Mass Spectrometry. The 24-h dietary recall data was entered into Xyris FoodWorks 10. All statistical analysis were conducted using Stata 17.0.

**Results:**

A total of 109 participants with complete 24-h urine samples were included in the analysis. From the 24-h urine collections, the median urinary excretion of sodium and iodine was 3,222 mg/24 h (25th, 75th percentile: 2516, 3969) and 112 μg/24 h (82, 134). The median estimated sodium intake from discretionary salt was 13% (25th, 75th percentile: 7, 22) of the total sodium intake or 366 mg/24 h (25th, 75th percentile: 186, 705).

**Conclusion:**

The total sodium intake was higher than the suggested dietary target of 2,000 mg/day. In this sample of healthy adults 18 to 40 years old, 13% of total sodium intake derived from discretionary salt. Discretionary salt is an additional source of iodine if iodised salt is used. Policies to reduce sodium intake is recommended to include a range of strategies to target discretionary and non-discretionary sources of salt and will need to take into account the contribution of iodine from discretionary salt intake.

## 1. Introduction

A high sodium intake is one of the leading dietary risk factors for mortality worldwide (1), accounting, globally, for 3.2 million deaths in 2017. Beyond the well-established relationship of high sodium intake and increased blood pressure (BP) and higher risk of cardiovascular diseases (2), other health outcomes such as chronic kidney disease and physical performance were also reported (3). One of the goals of the World Health Organization (WHO) “Global Action Plan for the Prevention and Control of Non-communicable Diseases 2013–2020” is to reduce population sodium intake by 30% (4). Sodium is naturally present in foods, but this makes a relatively small contribution to total sodium intake. Most dietary sodium comes from salt added to foods during manufacture, to foods prepared outside of the home (takeaways or restaurants), and to foods prepared in the home and at the table (discretionary salt). WHO recommends a range of strategies to reduce population sodium intake in the SHAKE Technical Package for Salt reduction, which include: food reformulation by setting sodium content goals for the food industry and restaurants; introducing front of pack labelling to inform consumers of food items high in sodium; and, consumer awareness campaigns that focus on the dangers of high sodium intake encouraging consumers to choose lower-sodium products and to use less discretionary salt (5).

The gold-standard method for measuring added or discretionary salt (sodium) is the lithium-tagged salt method developed in 1987 by Sanchez-Castillo et al. (6). This method involves fusing lithium carbonate to sodium chloride at a high temperature. Since the content of lithium in the human diet is negligible, the replacement of usual discretionary salt with lithium-tagged salt means that lithium-tagged salt can be used to objectively quantify sodium intake from discretionary salt. The method has a number of other advantages. Urinary recovery of lithium from the tagged salt reaches an equilibrium and plateaus after around 4 days of consumption (7). Thus, the duration of salt replacement required is relatively short. Furthermore, salt tagged with lithium does not alter the sensory quality of salt and the quantity of lithium consumed from the tagged salt is less than 0.4% of the therapeutic dose, making lithium-tagged salt both palatable and safe. This method has been used to estimate sodium intake from discretionary salt in the UK (8), Italy (9), Guatemala (10), Benin (11), Indonesia (12), and Denmark (13). Some of these studies were limited by small samples (fewer than 30 participants), specific population groups (i.e., mother-child pairs), with the most recent study published over 12 years ago.

In addition to contributing to sodium intake, salt, if iodised, can also contribute to iodine intake. Iodine deficiency is common, particularly in parts of the world, including New Zealand, where the soil naturally has a low content of iodine (14). As salt is a common staple in the diet, the addition of iodine to salt is a cheap, effective and recommended strategy to improve iodine intake (15). The effectiveness of iodised salt in reducing the prevalence of iodine deficiency is well documented (16). In New Zealand, iodised salt is added to most commercial breads (17), but is also used in the home. However, the amount of iodine and sodium derived from discretionary salt in the New Zealand diet is unknown. Like many countries in the world, sodium intake in New Zealand adults is high (18). It is important that any salt reduction strategies take into consideration the implications for iodine. The aim of this study was to estimate the proportion of total sodium that comes from added or discretionary salt in adults using the lithium-tagged salt method. A secondary aim was to identify other food sources of sodium and iodine.

## 2. Materials and methods

The study was approved by the University of Otago Human Ethics Committee (Health), reference number 19/168 and registered with the Australian New Zealand Clinical Trials Registry (ID: ACTRN12620000966998). The Strengthening the Reporting of Observational Studies in Epidemiology—Nutritional Epidemiology (STROBE-nut) epidemiology (19) was used in the reporting of the study (Checklist in Supplementary File).

### 2.1. Study design

**FIGURE 1:**
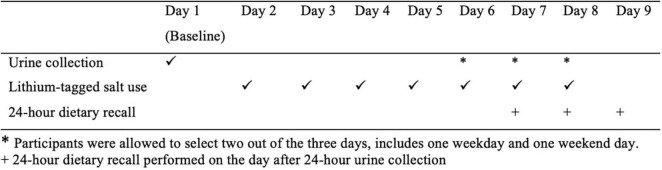
Overview of the study. *Participants were allowed to select two out of the 3 days, includes one weekday and one weekend day. + 24-h dietary recall performed on the day after 24-h urine collection.

This was a cross-sectional study of free-living adults. The study was undertaken over a 9-day period (Figure 1). Participants were asked to collect a baseline 24-h urine sample (Day 1) to establish their usual lithium excretion. From Day 2 to Day 8, participants replaced their normal table salt with the lithium-tagged salt, provided both in a pottle (i.e., 35 g) for cooking and in a saltshaker (i.e., 20 g) for use at the table. Water intake of participants was not restricted during this period as this does not affect sodium excretion (20). Participants were requested to follow their usual diet and use the lithium-tagged salt for cooking and at the table only during the study period, to store any food that was prepared at home before the study in the freezer, to use the lithium-tagged salt from the saltshaker when dining outside of the home, and to let the research team know if the amount of salt in either of the containers was low, so more could be provided. Regular contact with participants was established through text messages and phone calls to ensure that the study protocol was followed.

Lithium excretion in the urine is expected to plateau after 4 days of lithium-tagged salt consumption (7), thus another two 24-h urine samples were collected between Days 6 and 8; participants were allowed to choose the days for urine collection as long as there was one weekday and one weekend day. On the day after each of the final two 24-h urine collections, an in-person 24-h dietary recall was conducted. On the urine collection days, participants were advised to avoid vigorous exercise to prevent sodium and lithium loss through excessive sweating. Female participants were scheduled to take part in the study when they were not menstruating. Figure 1 outlines the study design.

### 2.2. Setting

Dunedin is located on the east coast of the South Island in New Zealand. This region has a temperate climate. Maximum daytime temperatures in summer are, generally, between 16 and 23°C and during wintertime, between 8 and 12°C (21). The study was conducted between October 2020 and November 2021. Data collection was paused between August 2021 and September 2021 due to COVID-19 restrictions.

### 2.3. Participants

Participants were recruited from Dunedin, New Zealand by advertisements placed at the University of Otago (University libraries, Unipol gym, Centre for Pacific Health, Māori Students’ Association), notice boards at supermarkets, Facebook, Instagram, word of mouth and e-mail invitations sent within the University of Otago email system. Participants were eligible if they were: between 18 and 40 years old; not pregnant; not planning a pregnancy; not breastfeeding; reported frequently eating meals prepared at home and using salt in cooking and/or at the table; did not have children under 18 years old living in the household; were not diagnosed with renal or heart disease; not taking diuretics or lithium-containing medication; and not taking kelp or other iodine-containing dietary supplements.

Interested participants contacted the study team by email. They were then screened over the phone and eligible participants were sent an electronic copy of the consent form. At the first study visit, the study protocol was verbally explained to the participants with time provided for questions. Participants willing to take part were required to sign the consent form and were reimbursed NZD100 (supermarket vouchers) to contribute to costs incurred related to taking part in the study.

### 2.4. Data sources and measurements

#### 2.4.1. Demographics

A basic demographic and salt-use questionnaire were administered using the REDCap (Research Electronic Data Capture) software (22). Ethnicity, income and education questions were asked using questions from the 2018 New Zealand census questionnaire (23).

#### 2.4.2. Blood pressure, height, and weight

Blood pressure was taken using an Omron Digital Blood Pressure Monitor HEM-907 (Omron Healthcare Co. Ltd., Kyoto, Japan) after participants were rested for at least 15 min (24). The BP monitor was set to automatically take two BP measurements at 30 s interval. Height (cm) and weight (kg) were measured twice using a SECA stadiometer and weighing scale model 876 (SECA GmbH, Hamburg, Germany). If the height and weight measurements were more than 0.5 units (i.e., cm and kg, respectively) apart, a third measurement was taken, and the closest two measurements were recorded. The BP, height and weight data were entered onto the REDCap software and the mean of the two measurements was used for each participant.

#### 2.4.3. Lithium-tagged salt

Lithium-tagged salt was produced at the University of Otago following the method described by Sanchez-Castillo et al. (6). A ratio of 9.1 g of lithium carbonate to 1 kg of sodium chloride was used in the preparation of the salt. Approximately 80 g of this mixture was placed in a 350 ml alumina crucible, heated to 900°C for 2 h in a muffle furnace, and allowed to cool overnight. The fused salt was broken down using a heat-treated chrome steel chisel and ground in a coffee grinder. The ground salt was then put through an Endecotts test sieve set to obtain a particle size of 100 to 850 μm. The lithium-tagged salt was produced in two separate batches of 1.5 and 3 kg. For every 150–200 g of lithium-tagged salt, 0.1 g sample of salt was removed to measure the lithium concentration determined by Inductively Coupled Plasma Mass Spectrometry (ICP-MS). The mean lithium content of the salt in first batch was 1.42 mg Li/g salt with a coefficient of variation (CV) of 3.0% (*n* = 10) and in the second batch was 1.36 mg Li/g salt with a CV of 3.8% (*n* = 15).

#### 2.4.4. Urine collection and analysis

The 24-h urine samples were collected according to the Pan American Health Organization (PAHO) protocol (25). Briefly, on the day of urine collection, the first void on the day was discarded with all subsequent voids collected in a 5 L plastic container including the first void of urine on the following morning. A log sheet was given to participants to note the start and end times of urine collection, as well as the time and reason for any missed voids. All urine samples were collected from the participants the next day and the volume measured. Three 10 ml aliquots of the urine were stored in −20°C until analysis.

Urine samples were thawed and mixed on the morning of analysis, and 500 μl aliquoted into the sampling cups. Urinary sodium was analysed using the Ion-Selective Electrode (26) method and urinary creatinine analysed through Jaffe reaction using alkaline picrate (27). Both analytes were measured on the Roche Cobas C311 system in the University of Otago, Department of Human Nutrition. Analysis of urinary lithium and iodine were conducted by the University of Otago, Department of Chemistry. Urine samples were thawed and mixed, and 200 μl aliquoted into the sampling cups. The samples were kept in a 4°C fridge until analysis. Urinary lithium and iodine was measured using ICP-MS (28). Pooled urine samples were analysed to ensure the precision of the instruments. The CV of the pooled urine samples for urinary sodium (*n* = 28), creatinine (*n* = 28), lithium (*n* = 14), and iodine (*n* = 14) were 1.9, 1.2, 3.8, and 0.8%, respectively.

#### 2.4.5. 24-h dietary recall

In-person multiple pass 24-h dietary recalls were conducted using methods based on that developed by the United States Department of Agriculture (USDA) (29). The first pass is the “quick list” where participants list the food items that were consumed the previous day. Participants are encouraged to associate the foods consumed with events or time of the day. Water consumption is also recorded at the end of the quick list. The second pass includes detailed descriptions of foods consumed, including cooking method, brand (if any), portion size, leftovers or second helpings. If the food was made at home, recipes of the ingredients are also recorded. Food models, pictures, and household measurements such as dinnerware, measuring spoons and cups are used to aid in the estimation of portion size. The food pictures used in this study are a standardised tool developed by the Department of Human Nutrition, University of Otago and routinely used in dietary surveys, including a nationwide cross-sectional study of adolescents (30). The final pass is a review with the participants where the researcher repeats the items recorded in the second pass to ensure accuracy of the recall. Additionally, to capture sodium intake from discretionary salt, participants were asked if they added salt when cooking or at the table. If participants reported using the saltshaker, they were asked to specify the number of shakes of salt they added to that food.

The 24-h dietary recalls were then entered into Xyris FoodWorks 10 (Xyris Pty Ltd., QLD, Australia) using the New Zealand FOODfiles 2018 (31). A complete nutrient profile was available for most foods, however, we had to amend nutrient values for items that were unavailable in FOODfiles 2018 as well as the iodine content of breads. When a food was unavailable in FOODfiles 2018, the nutrition information panel (NIP) of that food was obtained from the website of the food company or the website of a supermarket. A new food was then created in FoodWorks 10 using the nutrient information from a similar food in FOODfiles 2018 and information on the NIP. For example, a sugar-free version of a sports drink was absent in the FOODfiles 2018, a new food was created in FoodWorks 10 using the original version of the sports drink, then the energy, macro-nutrients and sodium values of the new food were amended to match the NIP of the sugar-free sports drink. The iodine content of bread in FOODfiles 2018 was out of date because it did not reflect the mandatory use of iodised salt legislated in New Zealand in 2009 (17), consequently, the iodine content of bread was updated using data from Plant and Food Research New Zealand (32).

We standardised the quantity of discretionary salt when food was in salted water (e.g., pasta, rice, vegetables), the sodium content of the food cooked in unsalted water was subtracted from the sodium content of same boiled food from the USDA FoodData, ^1^ and the difference in sodium was then converted to salt; the quantity of salt was then entered separately into FoodWorks 10. If the saltshaker was used in cooking or at the table, each shake was recorded as 0.06 g of salt. This weight was determined by averaging the weight of a shake of salt for three different researchers, 10 times.

A codebook which included standardised substitutions for food items was developed to ensure consistency between researchers when entering dietary data into FoodWorks 10. The 24-h dietary recalls obtained from the first 10 participants were entered in FoodWorks 10 by two researchers independently, utilising the codebook when required. There was less than 5% difference in total energy, sodium and iodine intakes between the 24-h dietary recalls entered by the two researchers, indicating good agreement between the researchers. Subsequently, all 24-h dietary recalls were entered by one researcher and a second researcher checked all entries to ensure that the food items and quantity were entered accurately.

#### 2.4.6. Classification of food groups

The food groups were classified according to the categories used in the 2008/09 New Zealand Adult Nutrition Survey (33). We collapsed some food groups considered to have a similar sodium and iodine content. For example, beef, veal, lamb, mutton, pork, and other meat were combined into one food group called red meat and red meat dishes. Additionally, a food group called sushi was created because the seaweed used in sushi is known to provide high levels of iodine (34). In total, there were 27 food groups (Table 4).

**TABLE 1:** Baseline characteristics of participants.

Characteristics	Total (*n* = 109)
Age (years), mean (SD)	26.0 (6.1)
**Sex, *n* (%)**	
Male	52 (48)
Female	57 (52)
Ethnicity*, *n* (%)	
Māori	8 (7)
Pacific peoples	6 (6)
Asian	20 (18)
European and other ethnicity	75 (69)
**Income (NZD/year), *n* (%)**	
Loss–$15,000	57 (52)
15, 001–$50,000	30 (28)
50, 000–$150,000	17 (16)
Did not respond	5 (5)
**Highest level of education, *n* (%)**	
Secondary school or less	44 (40)
Diploma/Bachelors	47 (43)
Postgraduate	18 (17)
**Blood pressure (mmHg), mean (SD)**	
Systolic	117 (12)
Diastolic	67 (8)
BMI (kg/m^2^), mean (SD)	24.3 (4.7)
**Salt used at home, *n* (%)**	
Iodised	66 (61)
Non-iodised	11 (10)
Mixture of iodised and non-iodised	18 (17)
Unsure	14 (13)
Baseline urinary iodine excretion (μg/24 h), median (25th, 75th percentile)	129 (92, 169)
Baseline urinary iodine concentration (μg/L), median (25th, 75th percentile)	60 (42, 113)

SD, standard deviation; BMI, body mass index; NZD, New Zealand dollars. *Prioritised ethnicity used, based on Stats NZ priority classification (62). Percentages reported may not total to 100 due to rounding.

**TABLE 2:** Urinary excretion of sodium, sodium intake from discretionary salt calculated from urinary lithium and urinary excretion of iodine.

	Males (*n* = 52)		Females (*n* = 57)		Total (*n* = 109)	
	Median (25th, 75th percentile)	Mean (SD)	Median (25th, 75th percentile)	Mean (SD)	Median (25th, 75th percentile)	Mean (SD)
Total sodium (mg/24 h)	3535 (3098, 4331)	3730 (1134)	2821 (2054, 3344)	2908 (1198)	3222 (2516, 3969)	3300 (1233)
Discretionary salt (sodium) (mg/24 h)	484 (201, 858)	687 (811)	308 (137, 528)	400 (394)	366 (186, 705)	537 (642)
Discretionary salt (sodium) (%)*	14 (7, 23)	19 (19)	12 (7, 20)	15 (13)	13 (7, 22)	17 (16)
Iodine (μg/24 h)	125 (109, 154)	130 (43)	88 (73, 112)	111 (102)	112 (82, 134)	120 (80)

*Sodium intake from discretionary salt as a proportion of total sodium.

**TABLE 3:** Estimated intake of energy, total sodium, sodium intake from discretionary salt and iodine from 24-h dietary recall.

	Males (*n* = 52)		Females (*n* = 57)		Total (*n* = 109)	
	Median (25th, 75th percentile)	Mean (SD)	Median (25th, 75th percentile)	Mean (SD)	Median (25th, 75th percentile)	Mean (SD)
Energy (kJ/day)	11060 (9623, 13266)	11501 (3012)	8684 (7028, 10616)	8902 (2476)	10040 (7945, 11718)	10142 (3027)
Total sodium (mg/day)	3658 (3011, 4673)	4191 (1989)	2939 (2519, 4089)	3515 (1653)	3430 (2737, 4630)	3838 (1844)
Discretionary salt (sodium) (mg/day)	568 (143, 1155)	949 (1270)	521 (206, 1074)	1034 (1493)	546 (173, 1074)	994 (1385)
Discretionary salt (sodium) (%)*	17 (6, 32)	21 (20)	18 (8, 37)	24 (22)	18 (7, 33)	23 (21)
Iodine (μg/day)	113 (73, 154)	125 (75)	83 (57, 104)	92 (61)	91 (60, 129)	108 (70)

*Sodium intake from discretionary salt as a proportion of total sodium.

**TABLE 4:** Number of participants who consumed food items in each food group and median sodium intake (mg/person/day) of participants who consumed food items in each food group, in total and by gender.

	Total (*n* = 109)		Males (*n* = 52)		Females (*n* = 57)	
Food groups	Number of participants who consumed food items, *n* (%)^a^	Sodium intake mg/person/day, median (25th, 75th percentile)^a^	Number of participants who consumed food items, *n* (%)^a^	Sodium intake mg/person/day, median (25th, 75th percentile)^a^	Number of participants who consumed food items, *n* (%)^a^	Sodium intake mg/person/day, median (25th, 75th percentile)^a^
Sushi	9 (8)	1071 (437, 1762)	4 (8)	1762 (542, 1800)	5 (9)	705 (437, 1437)
Pies and pasties	9 (8)	1038 (797, 1080)	5 (10)	938 (442, 1060)	4 (7)	1038 (1002, 1099)
Bread based dishes	32 (29)	941 (466, 1440)	18 (35)	949 (252, 1820)	14 (25)	909 (469, 1439)
Sausages and processed meats	38 (35)	855 (450, 1305)	23 (44)	867 (645, 1500)	15 (26)	675 (393, 1169)
Soups/stocks	15 (14)	645 (462, 1324)	6 (12)	600 (500, 2110)	9 (16)	690 (424, 1033)
Bread	91 (83)	494 (323, 818)	43 (83)	556 (342, 934)	48 (84)	460 (320, 708)
Savoury sauces and condiments	102 (94)	480 (183, 1028)	49 (94)	565 (198, 1014)	53 (93)	439 (183, 1337)
Fish/seafood	23 (21)	468 (243, 707)	7 (13)	275 (243, 697)	16 (28)	619 (309, 751)
Grains and pasta	93 (85)	412 (5, 1044)	45 (87)	498 (5, 1056)	48 (84)	321 (2, 1044)
Poultry and poultry dishes	49 (45)	310 (91, 576)	23 (44)	413 (111, 695)	26 (46)	264 (87, 469)
Other baked products	67 (61)	209 (84, 365)	29 (56)	152 (84, 323)	38 (67)	264 (87, 420)
Snack foods	40 (37)	208 (47, 428)	17 (33)	282 (119, 439)	23 (40)	125 (20, 425)
Cheese	70 (64)	204 (122, 400)	31 (60)	206 (138, 454)	39 (68)	198 (96, 385)
Breakfast cereals	33 (30)	196 (135, 282)	19 (37)	224 (163, 287)	14 (25)	147 (94, 232)
Red meat	36 (33)	188 (72, 600)	18 (35)	290 (73, 600)	18 (32)	148 (72, 489)
Eggs and egg dishes	49 (45)	157 (140, 225)	28 (54)	163 (148, 225)	21 (37)	150 (100, 163)
Potato, kumara, taro	55 (50)	133 (10, 441)	26 (50)	98 (8, 795)	29 (51)	168 (16, 389)
Vegetables	93 (85)	90 (15, 499)	45 (87)	84 (9, 590)	48 (84)	91 (16, 483)
Milk	80 (73)	68 (29, 138)	40 (77)	77 (23, 140)	40 (70)	61 (35, 112)
Butter and margarine	50 (46)	54 (33, 98)	23 (44)	70 (40, 109)	27 (47)	39 (25, 74)
Dietary supplements	12 (11)	49 (39, 79)	9 (17)	59 (39, 79)	3 (5)	39 (29, 59)
Dairy products	52 (48)	38 (18, 76)	23 (44)	39 (19, 74)	29 (51)	36 (16, 76)
Nuts and seeds	38 (35)	33 (3, 124)	17 (33)	69 (9, 159)	21 (37)	19 (1, 76)
Beverages	109 (100)	30 (18, 73)	52 (100)	34 (20, 84)	57 (100)	27 (15, 70)
Other sweets	80 (73)	9 (2, 36)	36 (69)	6 (1, 21)	44 (77)	13 (3, 46)
Fruit	60 (55)	4 (1, 10)	27 (52)	4 (1, 7)	33 (58)	3 (1, 13)
Fats and oils	31 (28)	0 (0, 0)	13 (25)	0 (0, 0)	18 (32)	0 (0, 0)

^a^In each food group, analysis only includes participants who have consumed food items in that food group. For example, 91 out of 109 participants consumed bread, sodium intake from bread is only calculated for the 91 participants have consumed bread.

### 2.5. Quantification of urinary and 24-h dietary recalls data

Incomplete urine collections were determined using the following criteria: self-reported of two or more missing void samples in 24 h; total urine volume less than 500 ml; or 24-h urinary creatinine excretion fell outside the reference range (4.0 to 17.0 mmol/day for females and 7.0 to 24.0 mmol/day for males) (35). All participants with complete baseline urine were included in the analysis. When both urines collected between days 6 and 8 were considered as complete collections, the mean of both 24-h urinary sodium, iodine, sodium intake from discretionary salt and proportion of sodium intake from discretionary salt was used for each participant. We found little difference (around 5%) in the medians of urinary sodium excretion between the two visits, therefore, if a participant only had one complete urine collected between days 6 and 8 (*n* = 13), data for the single sample was used.

Urinary volume was corrected to 24 h by dividing measured urine volume with participants’ reported collection time (in hours) and multiplied by 24 h. Urinary sodium (mmol/l), creatinine (mmol/l), iodine (ng/ml) and lithium (ng/ml) were calculated as concentration (mmol/l or ng/ml) multiplied by adjusted urinary volume (l). Urinary sodium and creatinine were converted to mg by multiplying its atomic weight (i.e., 22.99 for sodium and 113.12 for creatinine).

Sodium intake from discretionary salt (mg/day) was calculated based on lithium excretion. Lithium excretion at baseline allows for the correction of lithium intake not originating from the lithium-tagged salt (8). Baseline lithium excretion was subtracted from lithium excretion in each of the two 24-h urine collections between days 6 and 8. It was then divided by the concentration of lithium measured in the lithium-tagged salt (i.e., 1.42 mg/kg for salt batch 1 and 1.36 mg/kg for salt batch 2) and multiplied by 400 (1 g of sodium chloride contains 400 mg of sodium). One participant had a higher urinary lithium excretion at baseline than the urine collected between days 6 and 8, for this participant sodium intake from discretionary salt was considered as 0 mg/day. The proportion of sodium intake from discretionary salt was expressed as a proportion of total sodium intake.

For comparison, the estimates of energy (kJ/day), sodium (mg/day), iodine (μg/day), sodium intake from discretionary salt (mg/day) and sodium intake from discretionary salt as a proportion of total sodium intake (%) derived from 24-h dietary recalls were calculated in the same manner as the urine values (i.e., if two urines collected between days 6 and 8 were considered complete, energy intake per day will be the mean of the two diet recalls. But if only one complete urine between days 6 and 8 was available, energy intake will only be reported for the day with the complete urine).

The sodium and iodine intake of each food group were only calculated amongst participants who consumed food items from each food group because only some of the food groups were consumed by a participant on that day (i.e., a participant consumes 15 out of 27 food groups in a day).

### 2.6. Sample size

Based on a pilot study and other published studies, sodium intake from discretionary salt is estimated to be around 15%, therefore a sample size of 97 participants is needed to determine the proportion of dietary sodium from added salt with a precision of ± 7 and 95% CI. With an attrition rate of 15%, we will require a total sample size of 116 participants. For comparison, the sample sizes of published studies using lithium-tagged salt are as follows: UK (33 men, 50 women) (8); Italy (91 men, 91 women, 175 children) (9); Guatemala (9 sons, 9 mothers) (11); Benin (13 sons, 13 mothers) (11); Denmark (37 men, 50 women) (13); and Indonesia (15 mothers, 15 sons) (12).

### 2.7. Statistical analysis

All statistical analysis were conducted on Stata 17.0 (36). Medians, 25th and 75th percentiles were reported, as urinary sodium and iodine excretion are expected to be not normally distributed in the whole population (13, 37). The number of consumers in each food group was also calculated. Means and standard deviation are commonly reported for 24-h urinary sodium (18, 38), therefore were also reported.

## 3. Results

**FIGURE 2:**
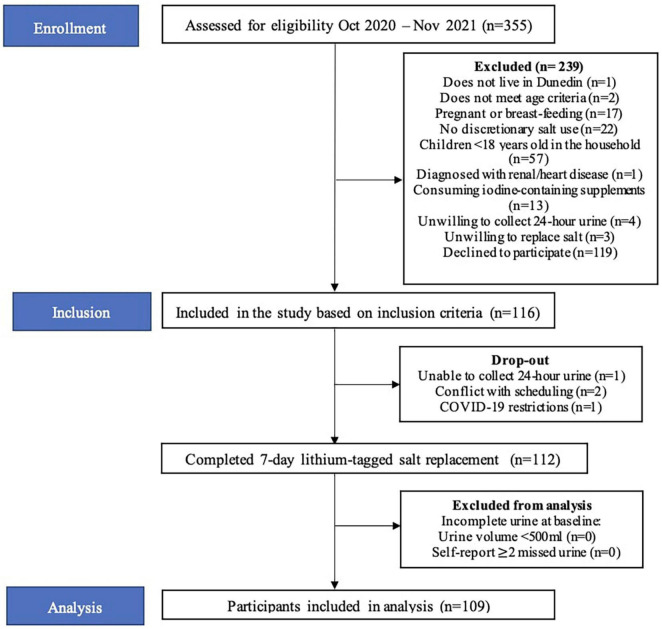
Participant flowchart.

In total, 335 people were screened over the telephone and 116 participants met the inclusion criteria and were included in the study (Figure 2). Four participants withdrew from the study for the following reasons: unable to collect urine (*n* = 1), conflicting schedules (*n* = 2) and COVID-19 restrictions (*n* = 1). Therefore, 112 participants completed the study. Participants who did not meet the criteria for complete baseline urine collections were not included (*n* = 3). The final sample size was 109.

Table 1 shows the baseline characteristics of the study participants. The mean age was 26.0 years and 48% of participants were male. The majority (69%) of participants were of “European and other ethnicity” and 52% were in the “Loss–NZD$15, 000” income category. A similar proportion of participants were in the completed secondary school or less (40%) and the tertiary studies (43%) category. Participants had a mean systolic BP of 117 mmHg, mean diastolic BP of 67 mmHg and mean body mass index of 24.3 kg/m^2^. All participants used discretionary salt and 61% of these participants used iodised salt at home. Baseline urinary iodine excretion was 129 μg/24 h and baseline urinary iodine concentration was 60 μg/L.

**FIGURE 3:**
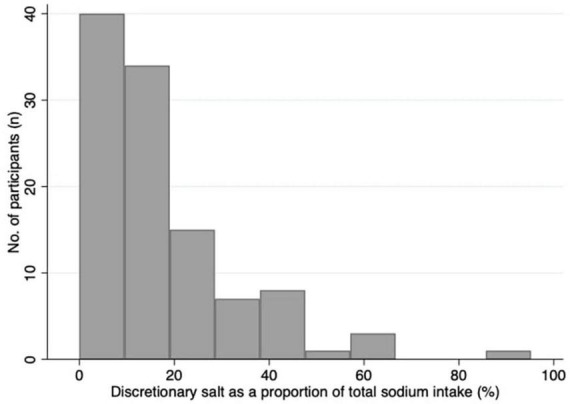
Histogram of the proportion of sodium intake from discretionary salt as a percentage of total sodium intake.

Table 2 presents the urinary excretion of total sodium, sodium from discretionary salt, total iodine, and sodium from discretionary salt as a proportion of total sodium. Overall, the participants’ median estimated sodium intake from discretionary salt was 13% (25th, 75th percentile: 7, 22) of total sodium intake or 366 mg in 24 h. The mean estimated sodium intake from discretionary salt intake was 17% of the total sodium intake or 537 mg/24 h. Estimated intake of sodium from discretionary salt in absolute terms and as a proportion of total sodium intake shows males consumed a median of 484 mg/24 h and 14% or mean of 687 mg/24 h and 19%. In females, the estimated intake of sodium from discretionary salt in absolute terms and as a proportion of total sodium intake was a median of 308 mg/24 h and 12% or a mean of 400 mg/24 h and 15%. Urinary excretion of total sodium was a median of 3,222 mg/24 h (mean: 3300 mg/24 h) and urinary iodine excretion was a median of 112 μg/24 h (mean: 120 μg/24 h). Figure 3 shows the number of participants and the proportion of sodium intake from discretionary salt. The majority of participants (85%) consumed less than 30% of their total sodium intake from discretionary salt. The maximum proportion of sodium intake from discretionary salt was 95% of total sodium intake.

Estimated intakes of energy, sodium, sodium from discretionary salt, sodium from discretionary salt as a proportion of total sodium and iodine from 24-h dietary recalls are presented in Table 3. The median intakes of energy, sodium, sodium from discretionary salt and iodine in all participants were 10,040 kJ/day, 3,430 mg/day, 546 mg/day, and 91 μg/day, respectively. Reported median intakes of energy, sodium and iodine are higher in males than in females. (11,060 kJ/day vs. 8,684 kJ/day; 3,658 mg/day vs. 2,939 mg/day; 113 vs. 83 μg/day, respectively). Based on the 24-h recalls, males and females reported similar intakes of sodium from discretionary salt as a proportion of total sodium, 17 and 18%, respectively.

Tables 4 and 5 show the sodium and iodine intake of participants who consumed food items in each food group, respectively. The tables also show the number of participants who consumed food items in each food group. The food group with the highest amount of sodium intake was sushi, followed by pies and pasties, bread-based dishes, sausages and processed meats, and soups/stocks (Table 4). The top five food groups contributing to iodine intake amongst consumers were sushi, eggs and egg dishes, bread, fish/seafood, and pies and pasties (Table 5). The food groups that were consumed by the most participants were beverages, savoury sauces and condiments, vegetables, grains and pastas, and bread.

**TABLE 5:** Number of participants who consumed food items in each food group and median iodine intake (μg/person/day) of participants who consumed food items in each food group, in total and by gender.

	Total (*n* = 109)		Males (*n* = 52)		Females (*n* = 57)	
Food groups	Number of participants who consumed food items, *n* (%)^a^	Iodine intake μg/person/day, median (25th, 75th percentile)^a^	Number of participants who consumed food items, *n* (%)^a^	Iodine intake μg/person/day, median (25th, 75th percentile)^a^	Number of participants who consumed food items, *n* (%)^a^	Iodine intake μg/person/day, median (25th, 75th percentile)^a^
Sushi	9 (8)	101 (57, 132)	4 (8)	119 (82, 142)	5 (9)	84 (57, 118)
Eggs and egg dishes	49 (45)	49 (41, 59)	28 (54)	51 (41, 74)	21 (37)	49 (27, 54)
Bread	91 (83)	38 (22, 70)	43 (83)	39 (27, 85)	48 (84)	34 (20, 61)
Fish/seafood	23 (21)	16 (6, 35)	7 (13)	21 (3, 61)	16 (28)	15 (7, 30)
Pies and pasties	9 (8)	8 (2, 15)	5 (10)	5 (2, 15)	4 (7)	9 (7, 15)
Milk	80 (73)	7 (3, 15)	40 (77)	8 (2, 16)	40 (70)	5 (3, 14)
Bread based dishes	32 (29)	6 (2, 13)	18 (35)	5.4 (1, 16)	14 (25)	5.8 (3, 13)
Grains and pasta	93 (85)	6 (1, 22)	45 (87)	7 (2, 23)	48 (84)	4 (0, 21)
Beverages	109 (100)	5 (4, 9)	52 (100)	7 (4, 10)	57 (100)	5 (3, 7)
Dairy products	52 (48)	4 (2, 9)	23 (44)	5 (2, 8)	29 (51)	4 (2, 10)
Other baked products	67 (61)	4 (1, 9)	29 (56)	4 (1, 6)	38 (67)	4 (1, 12)
Dietary supplements	12 (11)	3 (3, 5)	9 (17)	4 (3, 5)	3 (5)	3 (2, 4)
Soups/stocks	15 (14)	3 (2, 8)	6 (12)	3 (1, 14)	9 (16)	3 (2, 8)
Sausages and processed meats	38 (35)	3 (1, 8)	23 (44)	3 (1, 9)	15 (26)	2 (1, 6)
Vegetables	93 (85)	3 (1, 6)	45 (87)	3 (1, 7)	48 (84)	2 (1, 4)
Poultry and poultry dishes	49 (45)	2 (1, 6)	23 (44)	3 (1, 9)	26 (46)	2 (1, 5)
Red meat	36 (33)	2 (1, 6)	18 (35)	3 (2, 6)	18 (32)	2 (1, 5)
Cheese	70 (64)	2 (1, 4)	31 (60)	2 (1, 4)	39 (68)	2 (1, 3)
Potato, kumara, taro	55 (50)	1 (0, 3)	26 (50)	1 (0, 3)	29 (51)	0 (0, 3)
Savoury sauces and condiments	102 (94)	1 (0, 2)	49 (94)	1 (0, 2)	53 (93)	1 (0, 2)
Snack foods	40 (37)	1 (0, 2)	17 (33)	1 (0, 3)	23 (40)	1 (0, 2)
Nuts and seeds	38 (35)	1 (0, 2)	17 (33)	1 (0, 2)	21 (37)	0 (0, 1)
Other sweets	80 (73)	0 (0, 3)	36 (69)	0 (0, 3)	44 (77)	1 (0, 4)
Breakfast cereals	33 (30)	0 (0, 2)	19 (37)	1 (0, 3)	14 (25)	0.0 (0, 2)
Fruit	60 (55)	0 (0, 1)	27 (52)	0 (0, 1)	33 (58)	0 (0, 1)
Butter and margarine	50 (46)	0 (0, 0)	23 (44)	0 (0, 0)	27 (47)	0 (0, 0)
Fats and oils	31 (28)	0 (0, 0)	13 (25)	0 (0, 0)	18 (32)	0 (0, 0)

^a^In each food group, only includes participants who have consumed foods items in that food group. For example, 91 out of 109 participants consumed bread, iodine intake from bread is only calculated for the 91 participants have consumed bread.

## 4. Discussion

This is the first study in New Zealand to assess sodium intake from discretionary salt using the lithium-tagged salt method developed by Sanchez-Castillo et al. (6). Using the lithium-tagged salt method, we found that 13% of total sodium intake came from discretionary salt intake. Using 24-h dietary recall data, 18% of total sodium intake came from discretionary salt intake. We also found that bread was consumed by the majority of the participants (83%); the mandatory addition of iodised salt to bread means bread is a good source of iodine (38 μg/person/day) but also contributes to sodium (494 mg/person/day) intake. In contrast, egg and egg dishes provide a considerable amount of iodine, 49 μg/person/day, but a relatively low sodium intake of 157 mg/person/day in participants that consumed egg and egg dishes. Although consumed by fewer than 10% of participants, sushi contributed the highest amount of both dietary sodium and iodine.

In the few studies that have used the lithium-tagged salt method to determine sodium intake from discretionary salt (7–13), the proportion of sodium intake from discretionary salt to total daily salt intake has varied widely. In rural regions of low to middle income countries, such as Guatemala (10), Benin (11), and Indonesia (12), sodium intake from discretionary salt was between 50 and 70% of total sodium intake; this likely reflects diets where much of the food is prepared in the home. In countries where more of the food is purchased outside of the home, the proportion of total sodium intake coming from discretionary salt is closer to our findings. In 1987, the first lithium-tagged salt study conducted in the UK reported that the mean proportion of total sodium intake from discretionary salt was 16 and 17% for males and females, respectively (8). In Denmark, the reported median proportion of sodium intake from discretionary salt was 10% for males and 9% for females. Italy had a higher mean proportion of sodium intake from discretionary salt in males and females at 36 and 39%, respectively (13). This is perhaps due to Italians preparing more home-cooked meals than people in countries such as the UK (39). The lithium-tagged salt method requires participants to frequently consume discretionary salt, this may not represent the whole population, especially those who do not add salt in their cooking or at the table. Similarly, we recruited participants who self-reported using discretionary salt and we could have an overestimation of discretionary salt intake. However, in the 2008/09 New Zealand Adult Nutrition Survey, 98% of the participants reported using salt at home (33). Therefore, it is probably only a minority of the people in New Zealand who do not use discretionary salt. Our study sample also has a lower proportion of Māori participants as compared to the general population (7 vs. 17%) (40). However, for other major ethnic groups in New Zealand, our study ethnic make-up is similar to the general population. Namely, the study included 6% of Pacific peoples, 18% of Asians and 69% of European and Other and in the general population the proportion of each ethnicity is 8, 15, and 74%, respectively (40). Our study provides a general indication of discretionary salt use in New Zealand and does not aim to estimate discretionary salt use within each ethnic group.

Melse-Boonstra et al. used three methods to estimate the proportion of sodium intake from discretionary salt from nine mother-child pairs in Guatemala (10); the lithium-tagged salt method, a duplicate salt sample method, and the 24-h dietary recall. The study found that participants reported almost twice the amount of sodium intake from discretionary salt from the 24-h dietary recall compared to the lithium-tagged salt method, and the difference in reported sodium intake from discretionary salt was more than three times higher with the duplicate salt sample method compared to the lithium-tagged salt method. Although the difference we found in our study between the lithium-tagged salt method and 24-h dietary recall were less pronounced than in Melse-Boonstra et al., sodium intake from discretionary salt estimated from 24-h dietary recalls was still higher than the lithium-tagged salt method. To some extent, this could be due to the difference in the amount of salt added during cooking but not actually consumed. For example, the ingredients of a beef stew made with lithium-tagged salt was recorded in a recipe for the 24-h dietary recall, but there will be leftover gravy in the cooking pan or on the plate, which may not be consumed. Another reason for a higher sodium intake from discretionary salt found in 24-h dietary recalls could be overestimation because of visual misrepresentation. In our study, we used specific prompts to identify the use of discretionary salt (i.e., lithium-tagged salt) when recording recipes and foods eaten at the table in the 24-h dietary recalls. When participants report the amount of lithium-tagged salt used in a recipe, they would often refer to the measuring spoons. The smallest unit of measurement in the set of measuring spoons was ¼ teaspoon, and while this amount of salt seems to be a modest amount added to cooking, this still equates to 500 mg of sodium. Additionally, there may be a social desirability bias where participants were aware that the focus of the study was on sodium intake from discretionary salt. They may have reported using more lithium-tagged salt than they actually did to appear as more adherent to the study protocols. The PAHO and WHO do not recommend using 24-h dietary recalls or the duplicate salt sample method to measure sodium intake from discretionary salt (41).

Despite WHO’s recommendations, due to the large respondent burden of the lithium-tagged salt method, a range of other methods (including 24-h dietary recalls, duplicate salt sample) have been used to assess sodium intake from discretionary salt in some countries. A 2014 study conducted in three USA states (Alabama, Minnesota, and California) recruited 450 adult participants and estimated sodium intake from discretionary salt by collecting duplicate samples of discretionary salt during food preparation and at the table for 4 days (42). A total of 24-h dietary recalls were also conducted on the days after the duplicate salt samples were collected. Total sodium intake was estimated by adding sodium intake reported in the 24-h dietary recalls and a standardised calculation for table salt use based on the frequency and type of table salt. Total sodium intake was found to be 3,544 mg/day, of which 224 mg was from discretionary salt, representing 6% of total sodium intake. Estimating discretionary salt intake by collecting duplicate salt samples requires participants to replicate the amount of salt added to a dish they cook in a separate container. The amount of salt in the container is then weighed and based on the amount of food that participant reported consuming from that dish, discretionary salt intake is calculated. Another study, the 2015 China Health and Nutrition Survey, determined total sodium intake from three 24-h dietary recalls at the individual and household level. In addition, a household inventory of condiments was weighed at the beginning and at the end of each day to assess the amount of condiment use, including discretionary salt (43). Fifteen thousand participants were recruited from 11 provinces and three mega-cities in China. The study estimated that sodium intake from discretionary salt accounted for 59% of total sodium intake in the mega-cities and 67% in the provinces. A 24-h dietary recall relies on participants’ memory, ability to estimate portion sizes and willingness to report their food intake on the previous day (24). A study that collected 24-h dietary recalls and provided *ad libitum* intake to participants at a live-in research unit for 3 days where all food was weighed at the start and end of each day found that there is a tendency by participants to under-report unhealthy food intake (44). Finally, in South Africa, sodium intake from discretionary salt was assumed to be the difference between total sodium intake assessed by 24-h urinary sodium and sodium intake measured using 24-h dietary recalls collected from 325 adults (45). Sodium intake from discretionary salt was suggested to be between 33 and 46% of total sodium intake. Although estimating sodium intake from discretionary salt using 24-h dietary recalls or by collecting duplicate salt samples may be less burdensome for participants and researcher than using the lithium-tagged salt method, these methods are prone to reporting errors. Nevertheless, these studies further demonstrate the large variability of sodium intake from discretionary salt around the world and the need to identify sources of sodium in each country independently.

Dietary iodine is well absorbed and around 90% is excreted in the urine within 24 h (46). This means 24-h urine is a good biomarker for estimating recent iodine intake in a population (47). Notably in our study, when the participant’s usual salt was replaced by the lithium-tagged salt, which was not iodised, the median 24-h urinary iodine excretion for females was 88 μg/day; the recommended dietary intake is 150 μg iodine per day for New Zealand adults (48). We found that sodium intake from discretionary salt was 484 and 308 mg/day for males and females, respectively. In a previous study measuring the iodine content of iodised salt sold in New Zealand, the mean iodine concentration of 30 iodised salt samples was 50 mg iodine/kg salt (49). If the discretionary salt consumed by the participants was to be iodised at a concentration of 50 mg iodine per kg of salt, our results suggest that discretionary salt would contribute about 39 and 61 μg to daily iodine intake for females and males, respectively. The additional iodine intake from iodised salt is especially important for women of reproductive age as the requirements for iodine increases by more than 50% during pregnancy (50). Unfortunately, not all participants in this study reported using iodised salt at home. In an email from The Nielsen Company (Personal Communication), the sales of sea salt and pink Himalayan salt is more than 25% of total sales volume, indicating that the use of sea salt and pink Himalayan salt is popular in New Zealand, and these contain a negligible amount of iodine. The results of this study reinforce the “Eating and Activity Guidelines for New Zealand Adults” which suggest that if added salt is used, this salt should be iodised (51).

The use of iodised salt in bread contributes to both sodium and iodine intake. Since New Zealand introduced mandatory use of iodised salt in the production of commercials breads in 2009, iodine intake has improved in the population and bread is one of the main sources of iodine for people who consume bread (52, 53). Our study confirms this and in the participants who consumed bread, it contributed 494 mg/day and 38 μg/day to their sodium and iodine intake, respectively. However, there is a concern that those who do not consume bread (e.g., women), may not be getting enough iodine (54). Moreover, bread is one of the food products targetted to reduce salt content which will lower the amount of iodine obtained from bread if salt remains iodised at the same level (55). Other strategies to improve iodine intakes may be necessary. We found that eggs and egg dishes are a good source of iodine (49 μg/day) for consumers while not contributing a high amount of sodium (157 mg/day). The iodine content of an egg can be increased to 50 or 100 μg by adjusting the iodine content of the animal feed (56, 57). Consuming eggs with a higher iodine content has been shown to improve iodine intake in both iodine replete and deficient regions (56, 57). Furthermore, egg is a versatile food ingredient that can be cooked in many ways and easily incorporated into a person’s diet. Another good source of iodine is sushi; however, it also contains a large amount of sodium. We had to create a recipe for each type of sushi participants reported consuming, such as teriyaki chicken sushi and tuna mayonnaise sushi, as these food items are not available in FOODfiles 2018. The sodium in sushi primarily derives from sushi rice (available in FOODfiles 2018) and the sauces added to the filling (teriyaki, mayonnaise, etc.). For participants who consumed sushi, the median sodium intake from sushi is 1,071 mg/day. The amount of sodium consumed from sushi is similar to a previous study in New Zealand that analysed the sodium content of fast foods in the laboratory (58). The study found the sodium content of sushi per serving is 1,033 mg. The frequency of consumption of sushi in New Zealand is currently unknown. Iodine intakes can also be improved by increasing the concentration of iodine in iodised salt sold in New Zealand. For example, in Switzerland, iodine concentration in iodised salt was increased from 15 to 20 mg/kg and the median urinary iodine concentration increased by 23 and 80% in schoolchildren and in pregnant women, respectively (59).

Our study has several strengths. Sodium intake from discretionary salt was assessed using the gold standard, lithium-tagged salt method (41, 60). The low attrition and small number of incomplete 24-h urine collections shows high adherence to the study protocol. Our data was collected over a year and would have captured any seasonal difference in dietary intakes. We also reduced participant burden by collecting 24-h urine for 3 days instead of the 4 to 12 days required in similar studies (7, 8). A limitation of our study is consistent with all studies collecting 24-h urine. It is difficult to interpret incomplete urine collections and, currently, there is no consensus on the criteria used to ascertain complete urine collections (61). We have chosen to use a combination of three criteria (urinary creatinine, urinary volume and self-reported missed voids), which is a common practice in studies collecting 24-h urine (61).

## 5. Conclusion

The proportion of sodium intake from discretionary salt in our study is approximately 13% (25th, 75th percentile: 7, 22) of the total sodium intake. If this discretionary salt is iodised, it can provide an additional source of iodine. Total sodium intake is still high, well above the suggested dietary target of 2,000 mg/day, and needs to be reduced. A range of strategies targetting discretionary and non-discretionary salt intake may be employed based on the recommendations in the WHO’s SHAKE Technical Package for Salt reduction taking into consideration iodine nutrition.

## Data availability statement

The raw data supporting the conclusions of this article will be made available by the authors, without undue reservation.

## Ethics statement

The studies involving human participants were reviewed and approved by the University of Otago Human Ethics Committee. The patients/participants provided their written informed consent to participate in this study.

## Author contributions

NW obtained ethics approval, conducted the data collection and analysis, and drafted the original manuscript. RM and SS funding acquisition, obtained the ethics approval, provided the advice on data collection, supervised the data analysis, and reviewed and edited the manuscript. CC provided the statistical advice, supervised the data analysis, and reviewed and edited the manuscript. All authors have read and agreed to the published version of the manuscript.

## Footnotes

1
https://fdc.nal.usda.gov
